# The Two-Faced Role of Autophagy in Endometrial Cancer

**DOI:** 10.3389/fcell.2022.839416

**Published:** 2022-03-31

**Authors:** Tomohiko Fukuda, Osamu Wada-Hiraike

**Affiliations:** ^1^ Department of Obstetrics and Gynecology, JR Tokyo General Hospital, Tokyo, Japan; ^2^ Department of Obstetrics and Gynecology, The University of Tokyo, Tokyo, Japan

**Keywords:** autophagy, endometrial cancer, metformin, chemoprevention, chloroquine

## Abstract

Autophagy, meaning “self-eating,” is a cellular catabolic process that involves lysosomal degradation of cytoplasmic materials. Autophagy contributes to both quality control and energy supply of cells, which are associated with tumorigenesis and tumor development, respectively. Endometrial cancer (EC) is the most common gynecologic cancer, and its incidence is increasing. Although autophagy plays crucial roles in several types of cancer, such as pancreatic ductal adenocarcinoma, its role in EC has not been clearly demonstrated. Activation of the PI3K/AKT/mTOR pathway, which functions to suppress autophagy, is an initial step in type 1 endometrial carcinogenesis, whereas a loss-of-function mutation of *TP53*, which augments autophagy *via* p16 induction, is the main cause of type 2 endometrial carcinogenesis. Mutations in autophagy-related genes, including *ATG4C*, *RB1CC1/FIP200*, and *ULK4*, have been reported in EC; thus, an aberrant autophagy mechanism may be involved in endometrial carcinogenesis. Furthermore, the biguanide diabetes drug metformin, treatment with which enhances autophagy *via* AMPK-mediated mTOR inactivation, has been reported to reduce the risk of EC. These findings suggest that autophagy negatively regulates endometrial carcinogenesis, and autophagy inducers may be useful for chemoprevention of EC. In contrast, autophagy appears to promote EC once it is established. Consistent with this, treatment with chloroquine, an autophagy inhibitor, is reported to attenuate EC cell proliferation. Moreover, chemotherapy-induced autophagy triggers chemoresistance in EC cells. As autophagy has a tumor-promoting function, the combination of chemotherapy and autophagy inhibitors such as chloroquine could be a potent therapeutic option for patients with EC. In conclusion, autophagy plays a dual role in the prevention and treatment of EC. Therefore, targeting autophagy to prevent and treat EC requires diametrically opposed strategies.

## Introduction

The term “autophagy,” meaning “self-eating,” was initially used approximately 150 years ago (Ktistakis, 2017). Subsequently, Christian de Duve defined autophagy in 1963 as a cellular catabolic process that involves lysosomal degradation of cytoplasmic materials (Klionsky, 2008). Autophagy is divided into three types: macroautophagy, microautophagy, and chaperone-mediated autophagy (Mizushima and Komatsu, 2011). Of these, macroautophagy has been studied the most and is usually referred to as autophagy; hereafter, we refer to macroautophagy as autophagy. Microautophagy is a direct degradation machinery of lysosomes (Schuck, 2020). During microautophagy, invagination of the lysosomal membrane starts with the autophagy-related (ATG) proteins or in a Niemann-Pick type C (NPC)-dependent manner, followed by fission and degradation (Schuck, 2020). In contrast, chaperone-mediated autophagy does not involve membrane dynamics (Kaushik and Cuervo, 2018). Substrate proteins with a KFERQ motif are recognized by Hsc70 and transported into lysosomes by LAMP2A (Kaushik and Cuervo, 2018). However, unlike macroautophagy, the significance of microautophagy and chaperone-mediated autophagy is much less clear.

## Autophagy Machinery

### Overview of Macroautophagy (Autophagy)

**FIGURE 1:**
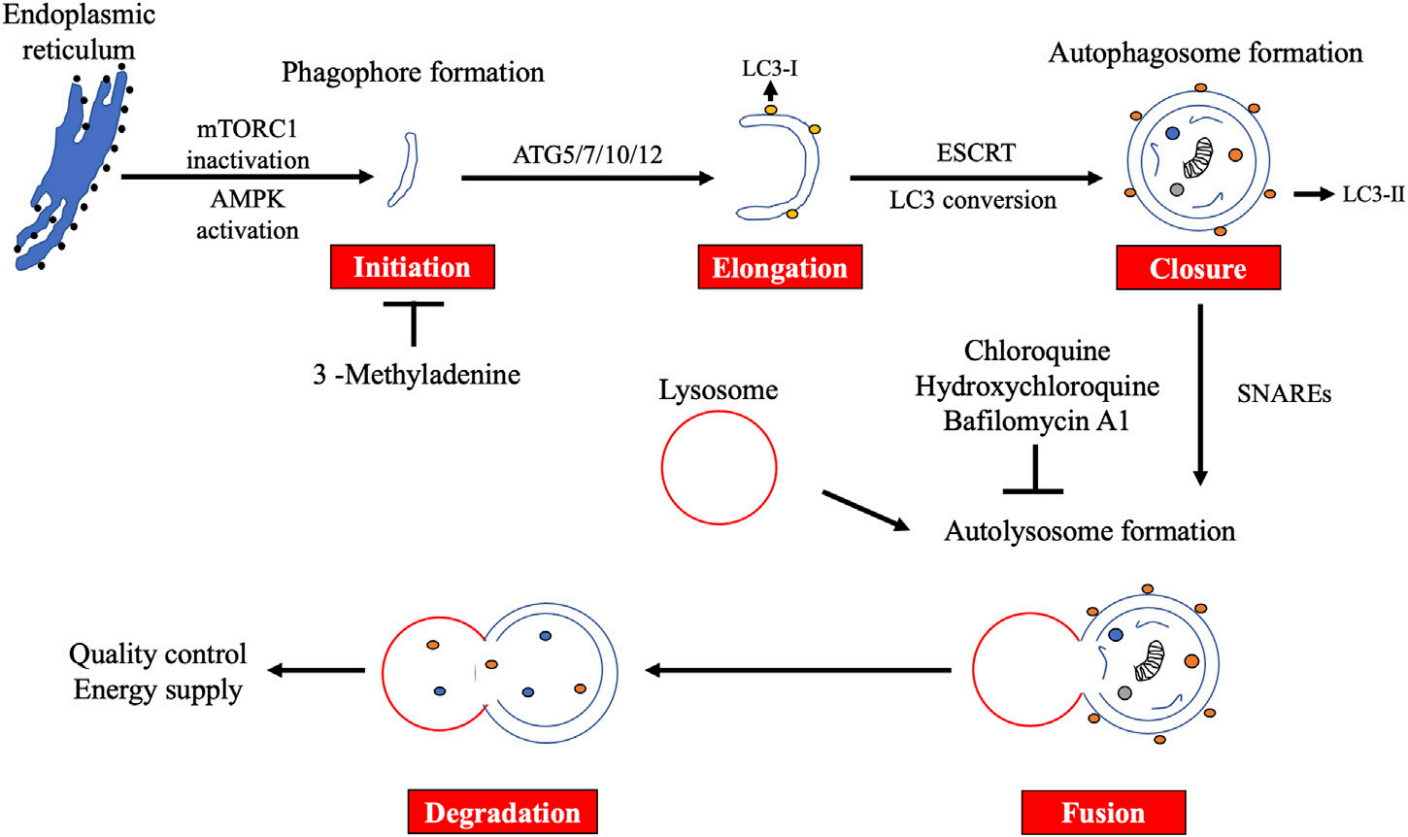
Overview of macroautophagy. Macroautophagy (autophagy) consists of five steps: initiation, elongation, closure, fusion, and degradation. Autophagy plays an important role in quality control and energy supply. 3-Methyladenine suppresses the initiation step, whereas chloroquine, hydroxychloroquine, and bafilomycin A1 block the fusion step.

Several stimuli, such as starvation and hypoxia, induce autophagy. This induction is mainly dependent on the mechanistic target of rapamycin complex 1 (mTORC1) inactivation or AMP-activated protein kinase (AMPK) activation (Mizushima and Komatsu, 2011). Autophagy is mediated by ATG proteins, which are evolutionarily conserved from yeast to mammals. To date, 43 *ATG* genes have been identified (Fukuda and Kanki, 2021). Fifteen of these (*ATG1-10*, *ATG12*, *ATG13*, *ATG14*, *ATG16*, and *ATG18*) are called core ATG genes because they are commonly utilized by both non-selective and selective autophagy. Autophagy consists of five steps: initiation, elongation, closure, fusion, and degradation (Figure 1) In response to the stimuli, the phagophore is initially formed near the endoplasmic reticulum (ER) and marked by ATG9 (Dunn, 1990). The initiation complex comprising UNC51-like kinase 1/2 (ULK1/2), ATG13, RB1CC1/FIP200, and ATG101 phosphorylates the class III phosphatidylinositol 3-kinase (PI3K) complex composed of ATG14, Beclin 1, VPS15, and VPS34, leading to the formation of a phagophore *via* phosphatidylinositol 3-phosphate (PI3P) generation. After recruitment of WIPI family proteins (WIPIs) and ATG2A/B, elongation of the phagophore begins. WIPI2 recruits ATG12 conjugates to ATG5 and ATG16L1 (ATG7 and ATG10) to elongate the phagophore. With ATG8 family proteins (ATG3, ATG7, LC3, and GABARAP subfamilies), the phagophore matures into the autophagosome. ATG8 family proteins recognize selective cargos, such as mitochondria (mitophagy) and ER fragments (ER-phagy) (Gatica et al., 2018). The edges of the autophagosome are closed by the endosomal sorting complex required for transport (ESCRT) machinery, followed by an acquisition of SNAP receptor (SNARE) proteins. The autophagosome fuses with lysosomes in a SNARE-dependent manner to form autolysosomes. Finally, the autolysosome degrades its contents.

### Autophagy Markers

Because autophagy is a dynamic process, monitoring it is not straightforward. To date, detection of accumulated autophagosomes is the gold standard for monitoring autophagy (Mizushima and Murphy, 2020). Autophagosomes can be detected biochemically as well as by microscopy. Biochemical detection involves immunoblotting of LC3B, which binds to the autophagosomal membrane (Mizushima and Murphy, 2020). LC3B can also be detected using immunocytochemistry (Ladoire et al., 2012). In addition, a GFP-LC3 reporter allows for direct detection of autophagosomes, although this does first require transfection. Autophagosomes can also be directly observed using electron microscopy. However, notably, autophagosome accumulation does not always equate to autophagy induction because reduced autophagosome consumption by lysosomes also results in autophagosome accumulation. To distinguish between these two phenomena, autophagy flux is measured in the absence and presence of lysosomal inhibitors, such as bafilomycin A1 or chloroquine (Mizushima and Murphy, 2020). LC3B resides in the cytosol as the LC3B-I isoform, and it is converted to the LC3B-II isoform on the autophagosomal membrane. Thus, quantification of the LC3B-II isoform reflects the number of autophagosomes. To assess autophagy flux, the amount of LC3B-II is compared with and without lysosomal inhibitors. If autophagy flux is enhanced, the difference between these groups will be high. However, if it is inhibited, the difference will be small. Moreover, autophagy flux can also be monitored by immunoblotting of SQSTM1/p62, which is a selective autophagy substrate (Mizushima and Murphy, 2020). When autophagy flux is enhanced, SQSTM1 expression is repressed in contrast to LC3B-II expression. Therefore, combinational immunoblotting of LC3B-II and SQSTM in the absence and presence of lysosomal inhibitors allows for more accurate assessment of autophagy flux.

### Monitoring Autophagy *In Vivo*

The autophagy markers mentioned earlier can be readily assessed *in vitro*. However, monitoring autophagy *in vivo* is challenging. To monitor autophagy flux, treatment with lysosomal inhibitors is necessary. Since lysosomal inhibitors have broad adverse effects *in vivo*, careful interpretation of the results is required. To avoid treatment with lysosomal inhibitors, several LC3 reporters have been developed and used in mice. For instance, transgenic mice expressing RFP-GFP-LC3 allows autophagy to be measured *in vivo* (Kaizuka et al., 2016). In addition, adeno-associated viruses that express mCherry-GFP-LC3 reporter can be injected intraventricularly in mice (Castillo et al., 2013). Unfortunately, monitoring autophagy in humans is much more challenging because these reporters cannot be applied to humans. Although no definitive method has been elucidated, immunohistochemical staining of LC3 in formalin-fixed paraffin-embedded tissue appears to be a promising approach (Ladoire et al., 2012). In contrast, frozen tissue is not recommended for immunohistochemistry because it contains lipid droplets that associate with LC3 but not with autophagosomes (Klionsky et al., 2021a). Several antibodies against LC3A and LC3B have been validated for immunohistochemical detection (Martinet et al., 2017). As it is not possible to distinguish between LC3-I and LC3-II isoforms and to exclude non-specific staining, the immunohistochemical findings need to be interpreted with a significant degree of caution. In line with this, a previous report has shown that cytoplasmic puncta over 1,000 nm in diameter are not consistent with autophagosomes (Ylä-Anttila et al., 2009). In addition, LC3A immunohistochemical structures can be classified as diffuse cytoplasmic, perinuclear, and stone-like (Sivridis et al., 2011). However, of these three structures, only the stone-like structure is associated with autophagosomes (Sivridis et al., 2011). Even if autophagosomes can be correctly stained, it is still difficult to monitor autophagy flux because lysosomal inhibitors are toxic to humans. Another target of immunohistochemical detection is SQSTM1. Similar to that indicated by immunoblotting, low levels of SQSTM1 in immunohistochemistry indicate enhanced autophagy flux. Several protocols for SQSTM1 immunohistochemical staining have been published recently (Martinet et al., 2017). Aside from LC3 and SQSTM1, there are no reliable autophagy markers for immunohistochemical detection.

## Endometrial Cancer

Endometrial cancer (EC) arises from the uterine endometrial epithelium, and it can invade the uterine myometrium and/or cervix. EC is the most common gynecologic cancer and the sixth most common female cancer worldwide, with an increasing incidence partly due to increase in obesity and longer lifespan (Sung et al., 2021). Since EC patients are often diagnosed at an early stage, with symptoms such as abnormal vaginal bleeding and lower abdominal pain, they have a relatively good prognosis compared with other gynecologic cancers; however, approximately 90,000 patients worldwide still die of EC per year (Sung et al., 2021). Once EC relapses, systemic chemotherapy is usually administered. However, the chemotherapeutic regimen for EC remains limited. Recently, immune checkpoint inhibitors have been used in EC patients with mismatch repair deficiency (dMMR) (Rousset-Rouviere et al., 2021). Moreover, the efficacy of the combination therapy of lenvatinib, which is a multi-receptor tyrosine kinase inhibitor, and pembrolizumab, which is an immune checkpoint inhibitor, has been confirmed, even in MMR-proficient EC patients (Marth et al., 2021). However, the cost-effectiveness of the combination therapy still remains an issue. Therefore, a new cost-effective molecular-targeted therapy is urgently needed for EC patients. Historically, EC has been classified into two types (Bokhman, 1983). Type 1 ECs mainly include low-grade endometrioid carcinomas, representing approximately 65% of all ECs. These tumors are triggered by unopposed excessive estrogen stimulation and develop from endometrial hyperplasia, the precursor lesions. They are often detected at an early stage due to abnormal vaginal bleeding and other early onset of symptoms. Molecularly, Type 1 ECs are associated with frequent mutations in *PIK3CA* and *PTEN* involving the PI3K/AKT/mTOR pathway and are often dMMR (Lax et al., 1998). Type 2 ECs comprise of high-grade endometrioid carcinomas and serous and clear cell carcinomas. These tumors are usually estrogen-independent due to the lack of estrogen and progesterone receptor expression, and they represent a more aggressive phenotype than type 1 tumors. Except for clear cell carcinomas, type 2 tumors are associated with *TP53* mutations and overexpression of HER2 (Llobet et al., 2009; Voss et al., 2012). According to The Cancer Genome Atlas, ECs can also be divided into four molecular subtypes: polymerase E (POLE)-mutant/ultramutated, microsatellite instability (MSI)-H, copy-number low, and copy-number high (Kandoth et al., 2013). MSI-H is consistent with dMMR, leading to a high tumor mutation burden (TMB). This classification is useful for the selection of immune checkpoint inhibitors.

## Mutations of Autophagy-Related Genes in EC

**FIGURE 2:**
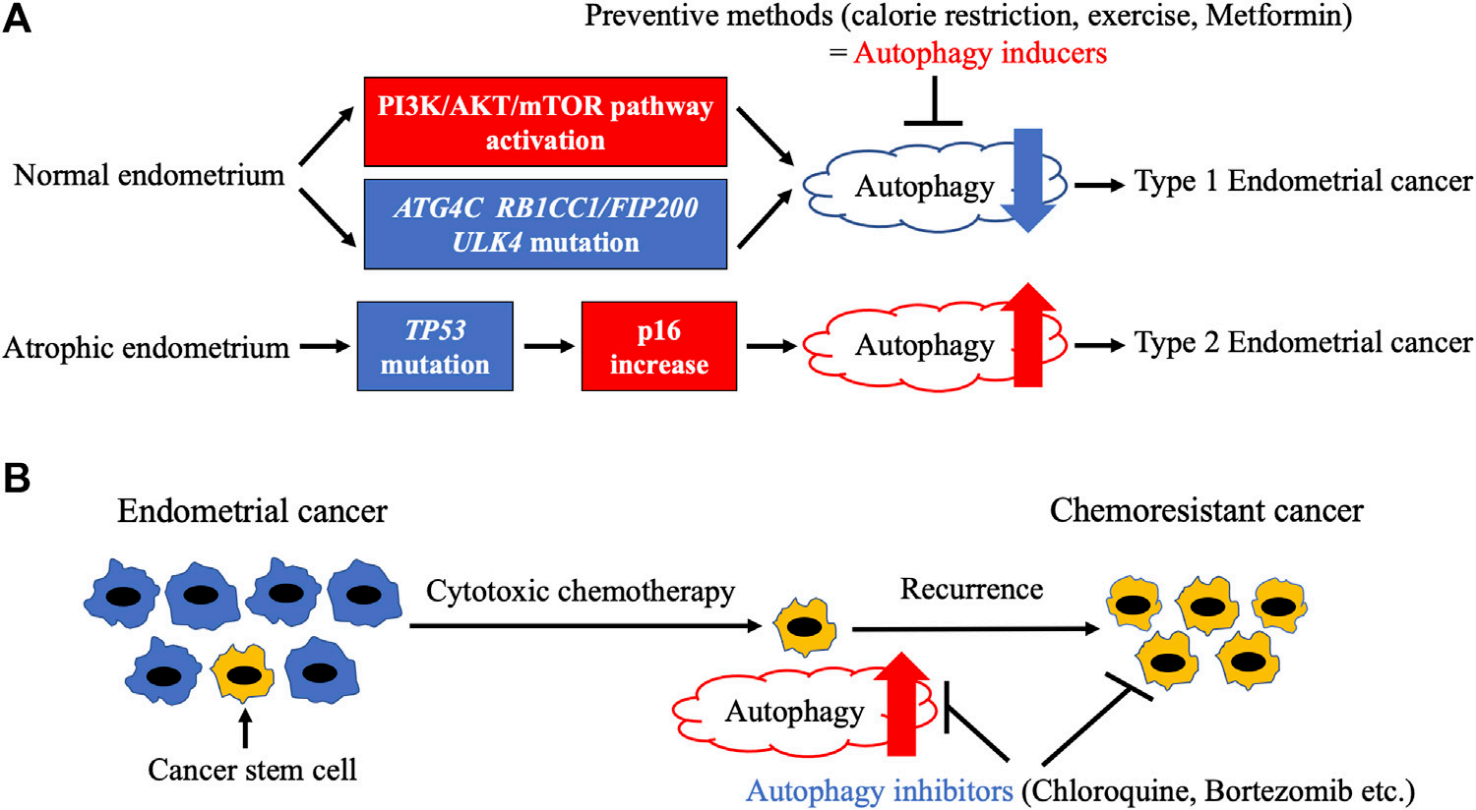
Relationship between autophagy and endometrial cancer. **(A)**. Endometrial carcinogenesis and autophagy. Frequent mutations of autophagy-related genes and activation of PI3K/AKT/mTOR pathway, which result in autophagy attenuation, are observed in endometrial cancer. Autophagy attenuation may lead to type 1 endometrial carcinogenesis, indicating a potential tumor-suppressive role of autophagy inducers such as calorie restriction, exercise, and metformin. In contrast, type 2 endometrial cancer develops from atrophic endometrium due to *TP53* mutation, which triggers an increase in p16 increase and autophagy. **(B)**. Endometrial cancer development and autophagy. Cytotoxic chemotherapy fails to kill cancer stem cells (CSCs) with increased autophagy, leading to cancer relapse. However, autophagy inhibitors can efficiently eliminate CSCs.

Although conditional knockout of *RB1CC1/FIP200* in the reproductive tract of female mice has been reported to result in infertility due to implantation failure (Oestreich et al., 2020), endometrial carcinogenesis has not been evaluated. In humans, an analysis of The Cancer Genome Atlas database regarding several types of cancer showed that mutations in autophagy-related genes were the most frequent in EC (Lebovitz et al., 2015). Significantly mutated genes included three autophagy-related genes—*ATG4C*, *RB1CC1/FIP200*, and *ULK4*—as well as *MTOR* (Lebovitz et al., 2015). RB1CC1/FIP200 and ULK4 are important for initiation, whereas ATG4C is involved in phagophore elongation. *MTOR* showed gain-of-function mutations by C1483F and S2215Y alterations. As mTORC1 inactivation is a major inducer of autophagy, these mutations may lead to autophagy attenuation. Interestingly, all ECs with mutations in autophagy-related genes were type 1 (Lebovitz et al., 2015), suggesting that autophagy plays a tumor-suppressive role in type 1 endometrial carcinogenesis (Figure 2A). However, both truncating (R1321*) and loss-of-function (S93L) mutations were observed in the *RB1CC1/FIP200* gene (Lebovitz et al., 2015), indicating an aberrant autophagy mechanism in type 1 endometrial carcinogenesis. Considering that parts of type 1 tumors display hyper (dMMR) or ultramutated phenotypes, further studies are needed to prove the significance of autophagy-related gene mutations in EC.

## Endometrial Carcinogenesis and Autophagy

As previously described, inhibition of autophagy may trigger type 1 endometrial carcinogenesis. It can hence be argued that autophagy inducers may be useful for the prevention of EC (Figure 2A). Among several strategies, weight loss with calorie restriction (CR) and exercise are initially attempted in obese women to prevent EC. Although CR is a potent inducer of autophagy in various metabolic tissues (Chung and Chung, 2019), its effect on endometrial autophagy has not been determined. In mice with induced endometriosis, CR dramatically stalled lesion growth along with autophagy induction (Yin et al., 2018), indicating that CR activates autophagy in orthotopic endometrium. The preventive role of exercise in endometrial carcinogenesis has been clarified (Tinelli et al., 2008; Friedenreich et al., 2010). Both exercise and CR enhance autophagy *via* mTORC1 inactivation in a number of tissues (Escobar et al., 2019). However, there is less evidence on the effect of exercise on endometrial autophagy. Further studies are needed to confirm whether CR and exercise induce autophagy in the endometrium. There are several chemopreventive agents for EC, including combined oral contraceptives (OCs), the levonorgestrel intrauterine system (LNG-IUS), bisphosphonates, and metformin. Combined OCs (COCs), which contain both estrogen and progesterone, have the strongest effect on the prevention of EC. At least 1 year of COC use reduces EC risk in proportion to the duration of their use (Collaborative Group on Epidemiological Studies on Endometrial Cancer, 2015). The effect persists for more than 30 years after their last use (Collaborative Group on Epidemiological Studies on Endometrial Cancer, 2015) (Iversen et al., 2017). Although estrogen or progesterone alone suppressed autophagy in the uteri of ovariectomized mice (Choi et al., 2014), autophagic modulation of human endometrium by COCs has not been determined. COCs have been reported to attenuate *BECN1* mRNA expression in the eutopic endometrium of patients with endometriosis (Waiyaput et al., 2021). However, it should be noted that Beclin 1 is not a specific autophagy marker. LNG-IUS is effective not only for the prevention of EC but also for the treatment of endometrial hyperplasia and early-stage EC (MacKintosh and Crosbie, 2018). Although a relationship between LNG-IUS and endometrial autophagy has not been reported, dienogest, another progestin, has been shown to induce autophagy in endometriotic cyst stromal cells in combination with estrogen *via* mTORC1 inactivation (Choi et al., 2015). Similar to LNG-IUS, dienogest also inhibited carcinogenesis in a mouse model of EC, but autophagy was not evaluated (Saito et al., 2016). Therefore, it is possible that LNG-IUS also suppresses endometrial carcinogenesis *via* autophagy induction. Bisphosphonates were initially developed as drugs for osteoporosis and are now also utilized to inhibit metastatic bone cancer (Mbese and Aderibigbe, 2021). A meta-analysis showed that bisphosphonates significantly reduced the risk of EC depending on their duration of use (Ou et al., 2016). Furthermore, zoledronic acid, the most frequently used bisphosphonate, directly suppressed invasion and sphere formation in EC cell lines *in vitro* (Muinelo-Romay et al., 2013). In addition, zoledronic acid induced autophagy in several cancer cell lines, including uterine cervical, prostate cancer, and glioblastoma (Lin et al., 2011; Wasko et al., 2011; Wang et al., 2014; Jiang et al., 2016). However, autophagy induction by zoledronic acid has not been detected in the endometrium. Metformin, a biguanide diabetes drug, is used to treat polycystic ovary syndrome (PCOS) patients with insulin resistance. Although metformin improves symptoms such as infertility and oligomenorrhea, its preventive effect on EC has not been confirmed in PCOS patients (Shafiee et al., 2014; Palomba et al., 2021). In contrast, metformin treatment significantly inhibited tamoxifen-induced endometrial changes in breast cancer patients (Davis et al., 2018). Metformin enhances autophagy mainly *via* AMPK-mediated mTORC1 inactivation. However, several other mechanisms have also been described (Lu et al., 2021). The mRNA expression levels of autophagy-related genes, including *ATG3,* were found to be reduced in the endometrium of PCOS patients compared with those in individuals with a healthy endometrium after metformin administration (Sumarac-Dumanovic et al., 2017). However, this needs to be interpreted with caution because ATG3 is not an autophagy specific marker. Another report indicated that metformin treatment notably augmented autophagy in the uteri of mice, as confirmed by immunohistochemical staining of LC3 and SQSTM1 (Wang et al., 2020). In addition, metformin simultaneously induced apoptosis (Wang et al., 2020). Further investigation is needed to clarify the relationship between metformin-induced autophagy and apoptosis. In summary, most preventive methods for typical type 1 ECs may commonly enhance endometrial autophagy. In contrast, type 2 ECs with a loss-of-function mutation of *TP53* showed a significant increase in the expression of *CDKN2A* mRNA (Kandoth et al., 2013), which has been shown to induce autophagy (Jiang et al., 2010; Budina-Kolomets et al., 2013). Thus, an aberrant autophagy mechanism may be related to endometrial carcinogenesis (Figure 2A).

## Autophagy and EC Development

Autophagy has been shown to play crucial roles in several types of cancer, including pancreatic ductal adenocarcinoma (PDAC) (Klionsky et al., 2021b). Mutations in *KRAS* and *TP53*, which are frequently observed in type 1 and type 2 ECs, respectively, are common in PDAC as well (Waddell et al., 2015). Increased autophagy has been reported in PDAC (Yang et al., 2011; Perera et al., 2015). Since the combined inhibition of autophagy and the ERK-MAPK pathway significantly suppressed PDAC *in vivo* (Bryant et al., 2019), a clinical trial involving combination of hydroxychloroquine and the MEK inhibitor trametinib is ongoing for PDAC (NCT03825289). The relationship between autophagy and EC is not as well understood as that with PDAC. Immunohistochemical staining of LC3A using endometrial samples indicated that autophagic activity was only observed in EC and atypical endometrial hyperplasia, as confirmed by the stone-like structures (Sivridis et al., 2011). Moreover, high counts of stone-like structures correlated with a poor prognosis in type 2 EC (Sivridis et al., 2011). This result is consistent with the notion that increased autophagy may trigger type 2 endometrial carcinogenesis. Although no other reports to date have directly assessed autophagy in clinical EC samples, the relationship between autophagy and EC has been investigated in EC cell lines and patient-derived xenografts. We previously reported that the autophagy inhibitor, chloroquine, suppressed the proliferation of EC cell lines (Fukuda et al., 2015), suggesting a tumor-promoting role of autophagy in EC. In addition, increased autophagy was related to cisplatin resistance in Ishikawa EC cells (Fukuda et al., 2015). Cisplatin has been reported to enhance autophagy *via* the PI3K/AKT/mTOR pathway inactivation in Ishikawa cells (Lin et al., 2017). Downregulation of HOTAIR, a long non-coding RNA, is another cause of cisplatin-induced autophagy in Ishikawa cells (Sun et al., 2017). Increased autophagy has also been observed in paclitaxel-resistant HEC-1A and JEC cells, and autophagy inhibition by chloroquine or *BECN1* knockdown has been shown to overcome resistance to paclitaxel (Liu and Li, 2015). Paclitaxel-induced autophagy has been reported to be dependent on both reactive oxygen species (ROS) generation and miR-218-mediated HMGB1 upregulation (Liu and Li, 2015; Sun et al., 2017). Cisplatin, a potent ROS inducer (Mirzaei et al., 2021) may enhance autophagy in part by inducing ROS in EC. DNA mismatch repair genes may also be important for chemotherapy-induced autophagy. The cytotoxic drug 6-thioguanine failed to induce autophagy in MSH2-knockout HEC59 EC cells (Zeng et al., 2007). Autophagy inhibition by *ATG5* shRNA enhanced 6-thioguanine-induced apoptosis in the parental HEC59 cells (Zeng et al., 2007). These results suggest that cytotoxic drugs trigger protective autophagy in EC (Figure 2B). Interestingly, increased autophagy was observed in Ishikawa-SP (side population) cells compared with Ishikawa-non-SP cells (Liu et al., 2020). In accordance with this, JEC spheroid cells exhibited higher levels of autophagy than JEC non-spheroid cells (Ran et al., 2017). Since SP and spheroid cells are considered to elevate stemness and cause tumor recurrence (Giannone et al., 2019), autophagy inhibitors may be effective at eliminating these cells, thereby leading to the suppression of EC relapse (Figure 2B). In addition to cytotoxic drugs, molecular-targeted agents can also modulate autophagy in EC through various mechanisms. Sorafenib, a multi-tyrosine kinase inhibitor, has been reported to trigger protective autophagy *via* activation of the JNK-MAPK pathway in EC cells (Eritja et al., 2017). Furthermore, sorafenib suppressed both EC cell line lung metastases and the growth of patient-derived xenografts in combination with chloroquine (Eritja et al., 2017). Bortezomib, a proteasomal inhibitor, blocked autophagy at the degradation step *via* the ERK-MAPK pathway activation in ES-2 EC cells (Kao et al., 2014). Similar to chloroquine, bortezomib augmented the cytotoxicity of cisplatin by blocking autophagy in ovarian cancer cells injected into mice (Kao et al., 2014). As previously described, mTORC1 inhibition is a major activator of autophagy. The mTOR inhibitor RAD001 has been reported to induce autophagy in Ishikawa and HEC-1A cells (Wang et al., 2016). In addition, RAD001 enhanced the cytotoxicity of paclitaxel in part *via* autophagy induction (Wang et al., 2016), indicating that excessive autophagy can also initiate EC cell death. Metformin, a chemopreventive drug for EC, augmented autophagy in Ishikawa cells (Takahashi et al., 2014). Inhibition of autophagy by 3-methyladenine attenuated metformin-induced apoptosis (Takahashi et al., 2014), suggesting that autophagy induction by metformin suppresses endometrial carcinogenesis as well as EC development. Cytotoxic autophagy in EC has also been confirmed with ABTL0812, which is a novel molecular-targeted drug (Felip et al., 2019; Muñoz-Guardiola et al., 2021). ABTL0812 induced autophagy *via* TRIB3-mediated AKT/mTOR pathway inactivation in EC cells (Felip et al., 2019; Muñoz-Guardiola et al., 2021). Moreover, ABTL0812 is currently in a phase 2 trial in patients with EC (NCT03366480). Autophagy modulation by estrogen has also been observed in EC cells. Estrogen enhanced autophagy *via* EIG121 induction (Deng et al., 2010), whereas it attenuated autophagy by promoting glutamine metabolism in EC cells (Zhou et al., 2019). Interestingly, EIG121 enhanced both autophagy and stemness in JEC cells (Ran et al., 2017), indicating that estrogen may trigger chemoresistance in ECs. Furthermore, estrogen receptor α (ERα) has been shown to form a complex with SQSTM1, followed by autophagic degradation in Ishikawa cells (Tsai et al., 2021). As estrogen activates multiple pathways, *in vivo* studies are needed to evaluate the effects of estrogen on EC. In contrast to chemoresistant EC cells, decreased autophagy was observed in progesterone-resistant Ishikawa cells (Zhuo et al., 2016). The PI3K/AKT/mTOR pathway activation and PTEN inhibition by miR-205 caused the autophagy decrease in Ishikawa cells (Liu et al., 2017; Zhuo and Yu, 2017). Metformin, which is an autophagy inducer, was also effective with progesterone-resistant Ishikawa cells (Zhuo et al., 2016). Therefore, autophagy inducers hold promise for the treatment of progesterone-resistant ECs. Finally, a number of different natural substances have been identified as inducers of autophagy in EC cells. Isoliquiritigenin and chrysin, two flavonoids, increased autophagy in EC cells (Wu et al., 2016; He et al., 2021). In addition, chrysin-induced autophagy was found to be dependent on ROS production (He et al., 2021). We have also reported that resveratrol triggered protective autophagy in Ishikawa cells (Fukuda et al., 2016). Furthermore, chloroquine enhanced resveratrol-induced apoptosis in Ishikawa cells (Fukuda et al., 2016). These results provide insight into the potential application of natural substances for the treatment of EC.

## Conclusion

Autophagy plays a dual role in the prevention and treatment of EC. Autophagy suppresses endometrial carcinogenesis, whereas it promotes the development of EC. Therefore, autophagy inducers and inhibitors may be effective in the prevention and treatment of EC, respectively. It should be noted that targeting autophagy to prevent and treat EC require diametrically opposed strategies. In order to confirm this notion, further *in vivo* studies and clinical trials are urgently needed in EC patients.
